# Ginkgo biloba extract suppresses hepatocellular carcinoma progression by inhibiting the recruitment of myeloid-derived suppressor cells through reduced CXCL1 secretion via SRC downregulation

**DOI:** 10.3389/fimmu.2025.1750890

**Published:** 2026-01-19

**Authors:** Zhiliang Xu, Siqin Liang, Xiaoxiang You, Anan Jin, Lei Mao, Fangyan Zhong, Tinghao Yuan, Jun He, Bo Yi, Ming Li, Qiang Tu

**Affiliations:** 1 Department of Hepatobiliary Tumor Surgery, Department of Interventional Therapy, Jiangxi Cancer Hospital, Nanchang, Jiangxi, China; 2 The Second Affiliated Hospital of Nanchang Medical College, Jiangxi Clinical Research Center for Cancer, Nanchang, Jiangxi, China; 3 First Clinical Medical College, Gannan Medical University, Ganzhou, Jiangxi, China; 4 Jiangxi Key Laboratory of Translational Cancer Research, Jiangxi Cancer Hospital of Nanchang University, Nanchang, Jiangxi, China; 5 Jiangxi Medical College, Nanchang University, Nanchang, Jiangxi, China; 6 2nd Abdominal Surgery Department, Jiangxi Cancer Hospital, Nanchang, Jiangxi, China

**Keywords:** ginkgo biloba extract, hepatocellular carcinoma, molecular docking, myeloid-derived suppressor cells, network pharmacology

## Abstract

**Background:**

Hepatocellular carcinoma (HCC) remains one of the leading causes of cancer-related mortality worldwide, characterized by poor prognosis and limited therapeutic efficacy. Ginkgo biloba extract (GBE) has demonstrated antitumor potential, yet its precise molecular mechanisms in HCC are not fully understood.

**Purpose:**

This study aimed to elucidate how GBE suppresses HCC progression and to explore its underlying molecular mechanisms.

**Methods:**

A subcutaneous HCC mouse model was established to evaluate the antitumor effects of GBE *in vivo*. Network pharmacology, molecular docking, and *in vitro* assays were integrated to identify and validate the core molecular targets of GBE.

**Results:**

GBE treatment significantly inhibited tumor growth and reduced myeloid-derived suppressor cells (MDSCs) recruitment within the tumor microenvironment. Network pharmacology identified proto-oncogene tyrosine-protein kinase (SRC) as a key target of GBE. Molecular docking revealed strong spontaneous binding affinity between active GBE components and SRC. *In vitro* experiments confirmed that GBE markedly downregulated SRC expression and CXCL1 secretion in HCC cells, whereas SRC overexpression reversed these effects. Clinical data further showed that SRC was upregulated in HCC tissues and correlated with poor prognosis and elevated MDSCs infiltration.

**Conclusion:**

GBE suppresses HCC progression by downregulating SRC expression, which consequently reduces CXCL1 secretion and limits MDSCs recruitment within the tumor. These findings highlight GBE as a promising adjuvant immunotherapeutic strategy for HCC.

## 1 Introduction

Globally, hepatocellular carcinoma (HCC) represents one of the most frequently diagnosed cancers and contributes substantially to cancer-related deaths (1). Its pathogenesis is driven by intricate interactions among diverse genetic alterations and multiple contributing factors, leading to rapid disease progression and unfavorable outcomes (2). Moreover, the global incidence and mortality of HCC continue to rise, with Eastern Asia and Africa experiencing the greatest disease burden (3). Histopathologically, primary liver cancers mainly include HCC and intrahepatic cholangiocarcinoma, with HCC representing nearly 85%–90% of total cases (4). Current mainstream therapeutic strategies for HCC comprise liver resection, local ablative therapies such as radiofrequency ablation, and transarterial chemoembolization, among others (5). However, their clinical applicability remains limited, and post-treatment outcomes are suboptimal due to high recurrence and metastasis rates (6). Hence, the development of new or adjunctive therapies with demonstrable efficacy is urgently required to improve patient outcomes and optimize HCC management.

Traditional Chinese Medicine (TCM) has gained increasing interest in cancer treatment due to its unique benefits, such as low cost and relatively mild side effects (7). Accumulating evidence indicates that TCM not only exerts therapeutic effects in HCC patients but also improves clinical prognosis. For example, a clinical study demonstrated that the Fuzheng Jiedu Xiaoji formulation delays HCC progression by specifically inhibiting the AKT signaling pathway (8). Another clinical investigation further found that adding Fufang Banmao Capsule to conventional treatment notably prolonged overall survival in individuals with HCC (9). Moreover, a recent mechanistic study provided deeper insights into the mode of action of Siwu Decoction (10). This formulation suppresses HCC progression by reshaping the tumor microenvironment, offering a novel theoretical basis and translational perspective for adjuvant therapeutic strategies. In summary, comprehensive and systematic investigations into the active components of TCM, their precise targets, and underlying molecular mechanisms are of great scientific significance for developing new TCM-based therapeutic approaches against HCC.

Ginkgo biloba extract (GBE) is a widely used standardized botanical preparation in traditional Chinese medicine, with core bioactive components including ginkgo flavonoids, terpenoid lactones, quercetin, luteolin, and kaempferol (11). Numerous studies have demonstrated that GBE exerts diverse biological effects, including anti-inflammatory, antitumor, antioxidant, and neuroprotective activities (12, 13). For example, GBE inhibits colorectal cancer cell proliferation and invasion by modulating TET2 expression (14), and pharmacological studies suggest it may suppress non-small cell lung cancer via the PI3K-AKT and MAPK signaling pathways (15). In China, GBE is incorporated into clinical guidelines and widely used as an adjuvant therapy for conditions such as diabetes, coronary heart disease, and cerebral insufficiency (16). Nevertheless, despite its broad therapeutic potential, studies on GBE specifically in HCC treatment remain limited. Existing evidence indicates that the combination of GBE and sorafenib shows good tolerability and an acceptable safety profile in patients with advanced HCC (17). However, a comprehensive understanding of GBE’s effects on HCC and its underlying pharmacological mechanisms remains limited. Elucidating the molecular mechanisms by which GBE inhibits HCC progression, identifying potential therapeutic targets, and evaluating its clinical applicability are of significant scientific value and may provide novel strategies for HCC prevention and treatment.

**Figure 1:**
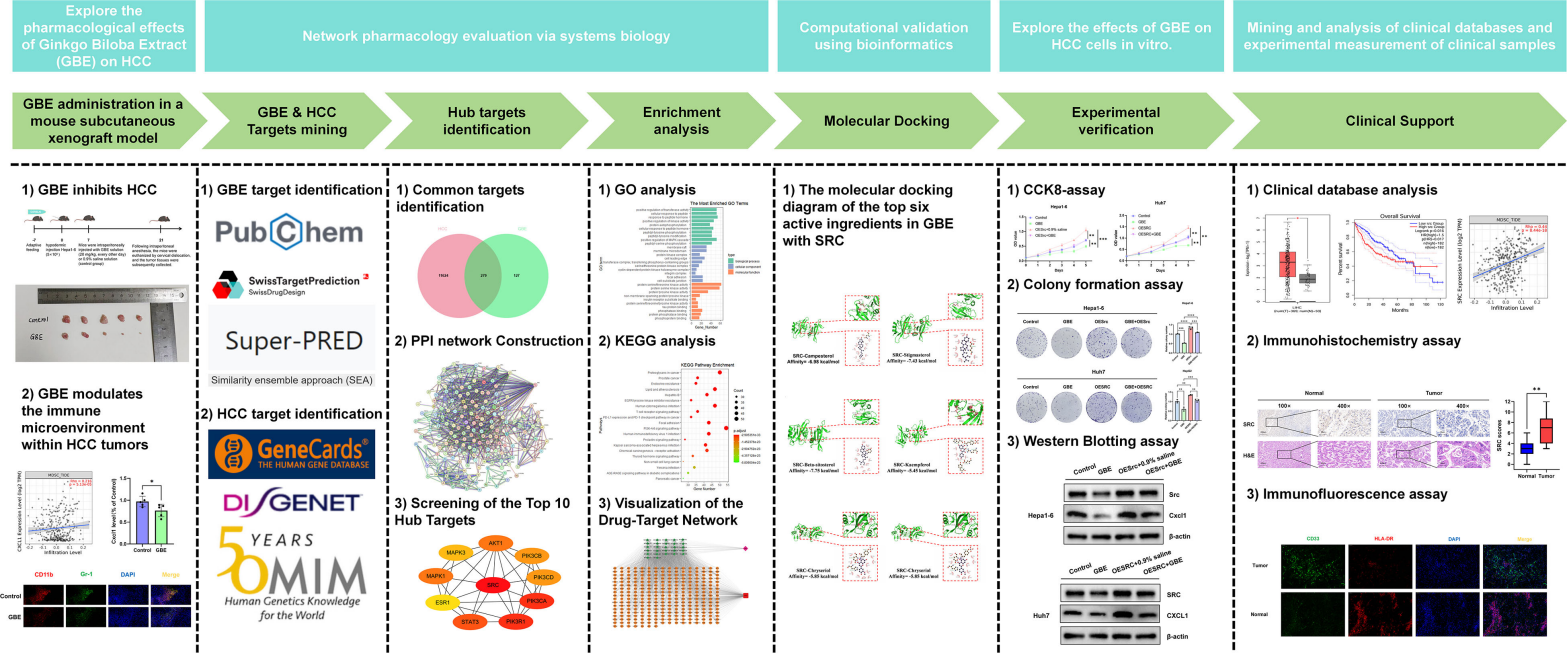
The flowchart of network pharmacology analysis and experimental validation of GBE in suppressing HCC progression.

This study employed an interdisciplinary strategy integrating network pharmacology and molecular docking, complemented by systematic investigations using animal models and cellular experiments. Network pharmacology, by combining concepts and methodologies from medicine, biology, and informatics, can map the complex interactions among drugs, targets, and diseases at a systems level, providing detailed predictions of a drug’s mechanisms of action (18). Molecular docking allows visualization and evaluation of ligand–protein interactions, providing insight into potential binding conformations and affinities (19). The integration of these approaches establishes a comprehensive research framework (**Figure 1**) to elucidate the molecular mechanisms and therapeutic potential of GBE in suppressing HCC from multiple levels and perspectives.

## 2 Network analysis and methodology

### 2.1 Screening of bioactive components in GBE and their potential therapeutic targets

In the Traditional Chinese Medicine Systems Pharmacology Database and Analysis Platform (TCMSP), a systematic search was conducted using the keyword “Yinxingye”. The obtained compounds were screened according to pharmacokinetic parameters—with oral bioavailability not less than 30% and a drug-likeness index of at least 0.18 (20)—to identify the bioactive components of GBE, resulting in a total of 27 compounds (**Supplementary Figure 1**). The canonical SMILES structures of these compounds were retrieved from the PubChem database and individually submitted to the SwissTargetPrediction and Super-PRED databases to predict potential targets. The predicted protein targets from these databases were uniformly converted to standard gene names using the UniProt database. Additionally, the Similarity Ensemble Approach (SEA) database was employed to supplement potential targets of the active components. Finally, the predicted targets from SwissTargetPrediction, Super-PRED, and SEA were merged, and after uniqueness processing (removal of duplicates), they were defined as the potential target set of GBE’s bioactive components.

### 2.2 Screening of HCC-related target proteins

Potential disease-related targets were retrieved by searching the keywords “hepatocellular carcinoma” in the following databases: GeneCards, DisGeNET and OMIM. The results from the four databases were consolidated, and after eliminating duplicates, all target identifiers were standardized to official gene symbols according to UniProt annotations.

### 2.3 Construction and topological analysis of protein-protein interaction network

The intersection between the predicted targets of GBE’s bioactive components and HCC-related disease targets was identified through Venn diagram analysis, yielding common core targets as potential therapeutic targets for GBE against HCC. Subsequently, PPI network was constructed using the STRING online resource with the following parameters: biological species set as “Homo sapiens”, minimum interaction confidence threshold > 0.9, and disconnected (isolated) target proteins hidden from the network to construct a high-confidence PPI network. Finally, the PPI results were visualized by Cytoscape, and a “bioactive component-target” relationship network for GBE was subsequently constructed. Core topological metrics of the network were computed using Cytoscape’s built-in analysis tools to obtain a more comprehensive understanding of bioactive compounds interacted with targets, as well as the interrelationships among target proteins.

### 2.4 GO and KEGG enrichment analysis

Using R tool and the bioinformatics package clusterProfiler, the overlapping core targets of GBE and HCC were analyzed for enriched biological functions and signaling pathways via Gene Ontology (GO) and Kyoto Encyclopedia of Genes and Genomes (KEGG) pathway analyses. The GO enrichment analysis aimed to systematically elucidate the Biological Processes, Cellular Components, and Molecular Functions in which these targets are involved (21). The KEGG enrichment analysis focused on identifying the major disease-related signaling pathways significantly enriched by these targets (22). Additionally, we employed the ggplot2 package to visualize key enrichment results, including significantly enriched GO terms, KEGG pathways, and their statistical metrics, to present the research findings clearly and intuitively.

### 2.5 Molecular docking

The three-dimensional structure model of the core gene proto-oncogene tyrosine-protein kinase (SRC) was first obtained from the UniProt database, while the SDF format files of the major active components of GBE were downloaded from the PubChem small molecule database. Molecular docking was conducted with the Molecular Operating Environment software suite. Finally, PyMOL molecular visualization software was employed to analyze the docking results in three dimensions, focusing on key molecular interactions such as hydrogen bonding and hydrophobic effects, and to generate high-quality 3D schematic diagrams of protein-ligand complexes.

### 2.6 Bioinformatics methods

Experimental procedures for C57BL/6 mice were illustrated using BioRender. Transcriptomic data obtained from Gene Expression Omnibus(GEO) were analyzed to determine differential gene expression in HCC cells exposed to GBE. Furthermore, the TIMER2.0 database was employed to analyze the association between gene expression and myeloid-derived suppressor cells (MDSCs) infiltration levels. Simultaneously, the Gene Expression Profiling Interactive Analysis (GEPIA2) database was employed to validate differences in the mRNA expression of core targets between normal liver tissues and HCC tissues, and to analyze the impact of their expression levels on patient overall survival. All websites used in this analysis are listed in **Table 1**.

**Table 1:** Basic information of the database used for the screening of GBE in the treatment of HCC.

Database	Website
TCMSP	https://www.tcmsp-e.com/
PubChem	https://pubchem.ncbi.nlm.nih.gov/
SwissTargetPrediction	http://swisstargetprediction.ch/
Super-PRED	https://prediction.charite.de/subpages/target_prediction.php
SEA	https://sea.bkslab.org/
GeneCards	https://www.genecards.org/
DisGENET	https://disgenet.com/
OMIM	https://www.omim.org/
jvenn	https://www.bioinformatics.com.cn/static/others/jvenn/example.html
STRING	https://cn.string-db.org/
Cytoscape	https://cytoscape.org/
RCSB Protein Data Bank	https://www.rcsb.org/
GEPIA2	http://gepia2.cancer-pku.cn/
TIMER2.0	http://timer.cistrome.org/
BioRender	https://www.biorender.com/
GEO	https://www.ncbi.nlm.nih.gov/geo/
LigPlot+	https://bioxm.software.informer.com/
R	https://www.r-project.org/
PyMOL	https://pymol.org/
Molecular Operating Environment	https://www.chemcomp.com/en/Products.htm
GraphPadPrism	https://www.graphpad.com/features
ImageJ	https://imagej.net/ij/
BioXM	https://cbi.njau.edu.cn/BioXM/

## 3 Experimental validation

### 3.1 Reagents and antibodies

Detailed information on reagents and antibodies is provided in **Supplementary Tables 1**, **2**. Preparation of GBE solution: Precisely weigh the GBE powder into a sterile centrifuge tube and add 0.9% normal saline to reach the desired concentration (1 mg/mL). Sonicate the mixture in an ice bath, with 30-second vortex mixing intervals between sonication cycles. After centrifugation and filtration, check the clarity of the solution. The freshly prepared GBE solution was used immediately for animal experiments.

### 3.2 Tissue specimens

Fresh HCC tissues and matched adjacent non-tumorous liver tissues were obtained from 40 patients who underwent surgical resection at Jiangxi Cancer Hospital between July 2021 and December 2022. The study protocol was reviewed and approved by the Ethics Committee of Jiangxi Cancer Hospital (Ethics approval ID: 2025ky187).

### 3.3 Cell lines and culture

Hepa1-6 (murine) and Huh7 (human) HCC cell lines were acquired from the Cell Bank of the Chinese Academy of Science. The identity of each cell line was authenticated using short tandem repeat profiling, and all lines were confirmed to be mycoplasma-free. Cells were maintained in Dulbecco’s Modified Eagle Medium containing 10% fetal bovine serum and 1% penicillin–streptomycin at 37 °C in a humidified incubator with 5% CO_2_.

### 3.4 Ethical approval for animal experiments

All animal experiments were performed in compliance with the Guidelines for the Care and Use of Laboratory Animals issued by the U.S. National Institutes of Health. Ten six-week-old male C57BL/6 mice were procured from Jiangsu Jicui Yaokang Biotechnology Co., Ltd. Under Specific Pathogen-Free (SPF) conditions, all mice were acclimated for one week with controlled environmental parameters (22 ± 1 °C, 55 ± 5% humidity, 12-h light/dark cycle) and had ad libitum access to diet and water. The mice were then randomly assigned into two groups: a treatment group (n = 5) and a control group (n = 5). Ethical approval for all experimental procedures was obtained from the Clinical Research and Animal Experimentation Ethics Committee of Jiangxi Cancer Hospital (approval number: 2025ky187).

### 3.5 Subcutaneous tumor-bearing mouse model and administration protocol

A total of 5×10^5^ logarithmically growing Hepa1–6 cells were implanted subcutaneously into the left flank of C57BL/6 mice. After inoculation, the mice were maintained under SPF conditions for 7 days. GBE solution (20 mg/kg dissolved in saline; injection volume: 0.4 mL) was intraperitoneally administered to mice assigned to the treatment group. The control group received 0.4 mL of normal saline under the same schedule. Over a two-week treatment course, mice received doses at 48-hour intervals, and tumor dimensions were evaluated every third day. Twenty-four hours after the final dose, mice were anesthetized with 1% pentobarbital sodium (50 mg/kg, i.p.) and euthanized by cervical dislocation. Tumor tissues were immediately and aseptically excised, weighed, and divided into portions—one fixed in 4% paraformaldehyde and the other snap-frozen in liquid nitrogen. All animal experiments were conducted in accordance with the Guidelines for the Care and Use of Laboratory Animals of the U.S. National Institutes of Health.

### 3.6 Enzyme-linked immunosorbent assay

Tumor tissues were homogenized according to the instructions of the ELISA kits, and CXCL1 levels were quantified using commercial kits from R&D Systems (Minneapolis, MN, USA). T cell suppression assays were performed according to a previously reported protocol (23). CD3^+^ T cells were isolated and activated as described above. Tumor-derived MDSCs were purified and co-cultured with CD3^+^ T cells at defined ratios (T cells: MDSCs = 1:1, 1:0.5, and 1:0.25). After 48 h of co-culture, supernatants were collected, and IFN-γ concentrations were quantified by ELISA using commercial kits according to the manufacturer’s instructions.

### 3.7 Other experiments

CCK8 and Western blot assays were conducted following the procedures we previously reported (24, 25). For the colony formation assay, 1,000 cells were seeded into 6 cm culture dishes and incubated for 14 days to allow colony formation. Colonies were first fixed using 4% paraformaldehyde following removal of the culture medium, and then stained with 0.5% crystal violet.

### 3.8 Plasmid construction and cell transfection

Two specific shRNA oligonucleotides targeting human SRC mRNA (shSRC-1: 5′-CCAGGCTGAGGAGTGGTATTT-3′; shSRC-2: 5′-GGTTTCAGAGGAGCCCATTTA-3′) and two targeting murine Src mRNA (shSrc-1: 5′-AGCGGCTGCAGATTGTCAATA-3′; shSrc-2: 5′-GACAATGCCAAGGGCCTAAAT-3′), along with a non-targeting control shRNA, were inserted into the pRNA-H1.1 vector to generate pRNA-H1.1-shControl constructs, pRNA-H1.1-shSRC-1, pRNA-H1.1-shSRC-2, pRNA-H1.1-shSrc-1 and pRNA-H1.1-shSrc-2. Overexpression plasmids (pcDNA3.1-SRC and pcDNA3.1-Src) were constructed by PCR amplification of human SRC and mouse Src coding sequences followed by subcloning into the pcDNA3.1(+) vector. Huh7 and Hepa1–6 cells were subsequently transfected with the corresponding plasmids using Lipofectamine 3000 as instructed by the manufacturer.

### 3.9 Multiple immunofluorescence assay

Cells were placed on poly-L-lysine–coated coverslips and maintained for 24 h. Samples were then fixed with 4% paraformaldehyde, permeabilized with 0.1% Triton X-100, and processed for immunofluorescence staining using fluorescent probes following routine protocols. Nuclear counterstaining was performed, and images were obtained using a Zeiss LSM 510 confocal microscope.

### 3.10 Immunohistochemistry and hematoxylin–eosin staining

Following deparaffinization and rehydration, sections were treated with 10% normal goat serum for blocking. The sections were then incubated with an anti-SRC primary antibody at 4 °C overnight (approximately 16–18 hours). Afterward, sections were incubated with a host-specific HRP-conjugated secondary antibody for 1 hour at room temperature, followed by a 30-minute treatment with Vectastain Elite ABC reagent (Vector Laboratories). Finally, the nuclei were counterstained with hematoxylin. The percentage scoring was performed based on the method reported by Huang et al (26). To ensure consistency and diagnostic accuracy, all H&E-stained sections were independently evaluated in a blinded manner by two experienced pathologists who were unaware of the group assignments. In cases of discrepancy between their evaluations, a joint review and discussion were conducted until a consensus was reached.

### 3.11 Statistical analysis

For statistical evaluation, differences between two groups were analyzed using Student’s t-test, while comparisons across multiple groups were performed with one-way or two-way ANOVA. All experiments were independently repeated at least three times. Data were analyzed with GraphPad Prism 9.0.0. Statistical significance was defined as follows: *, p ≤ 0.05; **, p ≤ 0.01; ***, p ≤ 0.001; ****, p < 0.0001.

## 4 Results

### 4.1 GBE inhibits HCC progression and reduces MDSCs recruitment

**Figure 2:**
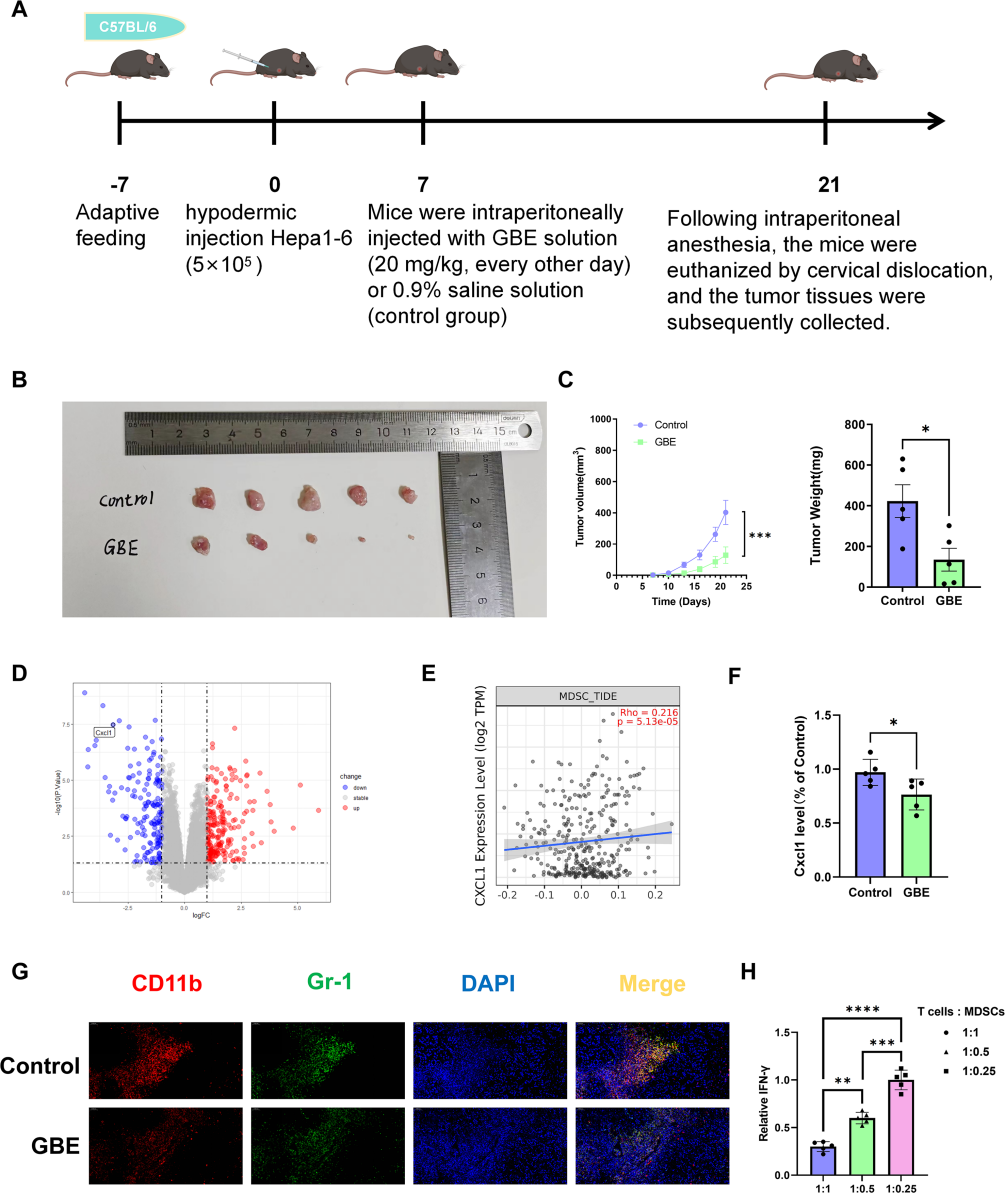
GBE suppresses HCC progression and reduces MDSCs recruitment. **(A)**Schematic diagram of the experimental workflow for the murine subcutaneous HCC model. This schematic was created using . **(B)** Diagram of excised tumors from the Control group (upper panel) and GBE-treated group (lower panel) at the study endpoint. **(C)** The tumor volume and weight of GBE-treated mice (20 mg/kg) and control mice (Normal Saline, 0.9%). Data are presented as mean ± SD. Statistical significance was determined by Student’s t-test: ***p < 0.001, *p < 0.05. **(D)** Volcano plot of GSE132575 dataset. **(E)** Correlation analysis via TIMER2.0 database confirmed a positive association between CXCL1 expression and MDSCs (*p < 0.05). **(F)** ELISA was used to detect the expression of Cxcl1 from tumor tissue homogenates in control and GBE-treated groups(*p < 0.05). Experiments were performed three times and data are presented as mean ± SD. **(G)** The immunofluorescence images of tumor tissues stained for Gr-1 (green) and CD11b (red). Co-localization of Gr-1 and CD11b (yellow) identifies MDSCs. Nuclei are counterstained with DAPI (blue). Scale bar: 50 μm. **(H)** T cell suppression assay using murine tumor–derived MDSCs. T cells were co-cultured with MDSCs at different T cells-to-MDSCs ratios (1:1, 1:0.5, and 1:0.25), with the 1:0.25 group serving as the control condition with minimal MDSCs-mediated suppression. The production of IFN-γ by T cells was assessed and normalized to the control group. Increasing proportions of MDSCs resulted in a progressive reduction of IFN-γ production, indicating a dose-dependent suppressive effect of MDSCs on T cell function. Data are presented as mean ± SD. Statistical significance was determined using one-way ANOVA followed by appropriate *post hoc* tests. P values are indicated as **P < 0.01, ***P < 0.001, and ****P < 0.0001. HCC, Hepatocellular carcinoma; GBE, Ginkgo Biloba Extract; MDSCs, myeloid-derived suppressor cells; IFN-γ, interferon-γ.

To investigate the effect of GBE on HCC, we established a murine subcutaneous HCC model (experimental workflow detailed in **Figure 2A**). The results demonstrated that GBE treatment significantly inhibited tumor progression in tumor-bearing mice, as evidenced by reduced tumor volume and decreased tumor weight (**Figures 2B, C**). To further elucidate this phenomenon, we conducted bioinformatic analysis of the GEO dataset (GSE132575) and found that Cxcl1 expression was significantly downregulated in the GBE-processed group compared to the primary HCC group (**Figure 2D**). Given that CXCL1 serves as a key chemokine ligand for MDSCs recruitment (27), we utilized the TIMER2.0 database to validate a positive correlation between CXCL1 expression and MDSCs recruitment levels (p < 0.05) (**Figure 2E**). Furthermore, ELISA results confirmed that Cxcl1 secretion was significantly reduced in tumor tissues from GBE-treated mice compared to the control group (**Figure 2F**). Immunofluorescence staining showed reduced MDSCs recruitment in tumor tissues after GBE treatment (**Figure 2G**). To functionally validate these cells, a T cells suppression assay was performed. Murine tumor-derived MDSCs significantly inhibited T cells effector function, as indicated by decreased IFN-γ production in co-culture (**Figure 2H**). These results suggest that GBE can inhibit HCC progression and reduce MDSCs recruitment *in vivo*.

### 4.2 Analysis of potential targets of GBE against HCC based on network pharmacology

**Figure 3:**
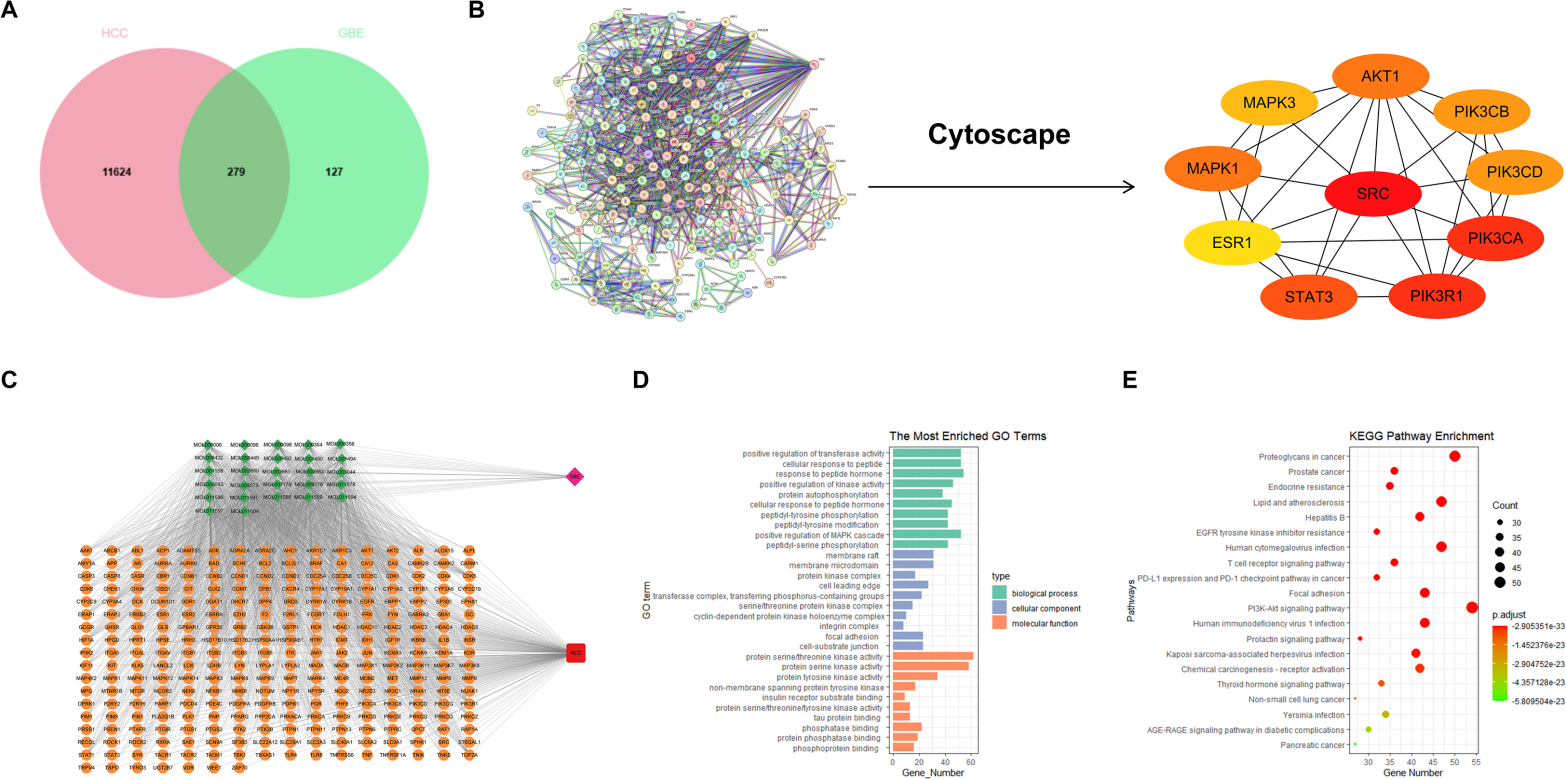
Network pharmacology analysis of potential targets of GBE on HCC. **(A)**Venn diagram (The intersection targets of GBE and HCC). **(B)** PPI network of the overlapping targets between GBE and HCC. Nodes represent proteins, and edges represent functional or physical associations between them. **(C)** Drug-intersection targets network. (Green diamonds represent chemical compounds, labeled with their MOL IDs from the TCMSP database; circles represent overlapping targets.) **(D)** GO analysis of intersection targets. **(E)** KEGG analysis of intersection targets. HCC, Hepatocellular carcinoma; GBE, Ginkgo Biloba Extract; TCMSP, Traditional Chinese Medicine Systems Pharmacology; GO, Gene Ontology; KEGG, Kyoto Encyclopedia of Genes and Genomes; PPI, Protein-Protein Interaction;.

Based on the TCMSP database, we identified 27 bioactive components of GBE. A total of 406 potential targets of these components were predicted using the SwissTargetPrediction, Super-PRED, and SEA databases. By integrating four disease databases (GeneCards, DisGeNET, DrugBank, and OMIM), a total of 11,903 HCC-related targets were identified. Venn diagram analysis revealed 279 common targets between GBE and HCC (**Figure 3A**). The intersecting targets were used to construct a PPI network, with disconnected nodes hidden. The resulting network consisted of 276 nodes with 313 expected edges and 1136 actual edges. The top 10 genes ranked by degree value were selected for further analysis and visualized using Cytoscape (**Figure 3B**). Notably, CXCL1 was not in 279 common genes. Given that SRC exhibited the highest degree value in the network, it was identified as the core target gene for subsequent mechanistic studies. A compound-target interaction network was also constructed (**Figure 3C**). By analyzing network topology features, including degree, betweenness, and closeness centrality, the top 10 bioactive compounds were determined (**Table 2**). The GO and KEGG enrichment results showed that these overlapping targets were markedly enriched in pathways such as lipid metabolism and atherosclerosis, serine/threonine kinase activity, and the PI3K-Akt signaling pathway (**Figures 3D, E**). Previous transcriptomic analysis showed that GBE treatment significantly reduced CXCL1 expression. Previous studies have reported that pathways enriched in SRC-associated signaling are involved in the regulation of the chemokine (28, 29). Therefore, CXCL1 was selected as a functionally relevant downstream effector of SRC. These findings provide a theoretical foundation for subsequent molecular docking and experimental validation.

**Table 2:** Top 10 targets information of PPI network.

MOL ID	Compound	Degree	OB	DL	Betweenness centrality	Closeness centrality
MOL005043	Campesterol	92	37.58	0.71	0.0362	0.4324
MOL000449	Stigmasterol	89	43.83	0.76	0.0197	0.4264
MOL000358	Beta-sitosterol	87	36.91	0.75	0.0137	0.4240
MOL000422	Kaempferol	87	41.88	0.24	0.0226	0.4252
MOL003044	Chryseriol	86	35.85	0.27	0.0128	0.4217
MOL000098	Quercetin	86	46.43	0.28	0.0108	0.4194
MOL002881	Diosmetin	82	31.14	0.27	0.0109	0.4205
MOL005573	Genkwanin	82	37.13	0.24	0.0205	0.4183
MOL001558	Sesamin	81	56.55	0.83	0.0187	0.4160
MOL001494	Mandenol	81	42.00	0.19	0.0094	0.4160

### 4.3 Molecular docking validation of GBE with SRC

**Figure 4:**
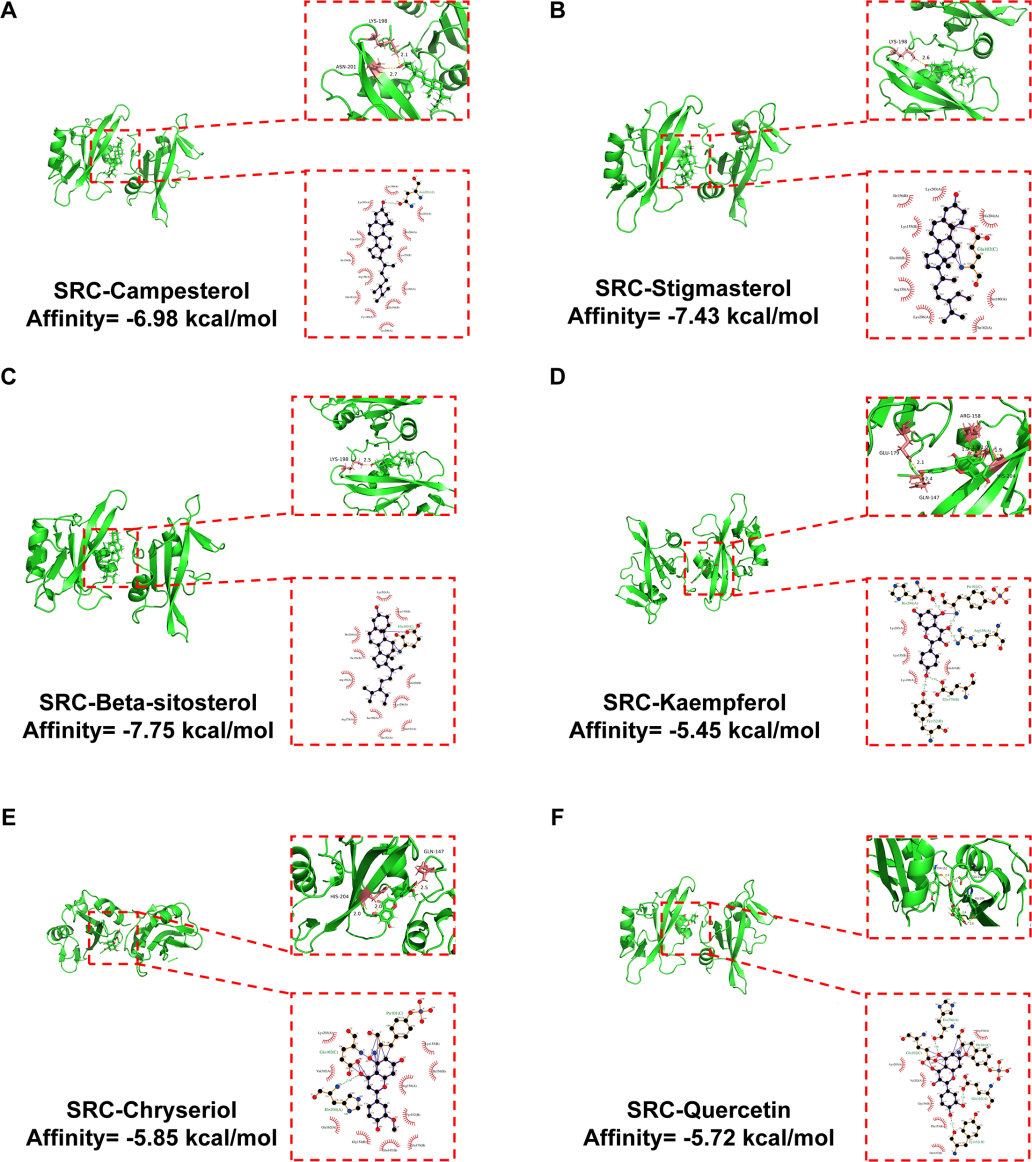
2D and 3D molecular docking between the SRC protein and the top six active compounds. **(A)** Interaction diagram between SRC and Campesterol. **(B)** Interaction diagram between SRC and Stigmasterol. **(C)** Interaction diagram between SRC and Beta-sitosterol. **(D)** Interaction diagram between SRC and Kaempferol. **(E)** Interaction diagram between SRC and Chryseriol. **(F)** Interaction diagram between SRC and Quercetin. SRC, proto-oncogene tyrosine-protein kinase.

To assess the interaction strength between GBE’s bioactive compounds and SRC, this study selected the top six compounds ranked by degree values from **Table 2** for molecular docking analysis with SRC. **Figure 4** displays the optimal binding conformations, hydrogen bond interactions, and the minimum binding energies between ligands and receptor obtained from 20 independent docking simulations. Analysis revealed that Campesterol bound to residues LYS-198 and ASN-201 of SRC; Stigmasterol and Beta-sitosterol both utilized LYS-198 as a key binding site; Kaempferol formed hydrogen bonds with GLU-179, GLN-147, ARG-158, and HIS-204; Chryseriol interacted with HIS-204 and GLN-147; and Quercetin targeted TYR-152, GLU-162, and HIS-204. All docking binding energies ranged from −5.45 to −7.75 kcal/mol, meeting the threshold for potential binding activity (<−5.0 kcal/mol). Notably, Beta-sitosterol exhibited the strongest binding capacity (−7.75 kcal/mol), consistent with previous reports that Beta-sitosterol inhibits HCC progression by potentially targeting SRC (30). These data indicate that the bioactive components of GBE exert anti-HCC effects by forming 1–6 specific hydrogen bonds with SRC, although this mechanism of action requires further validation through subsequent experiments.

### 4.4 SRC acts as a core factor of GBE in suppressing HCC progression​

**Figure 5:**
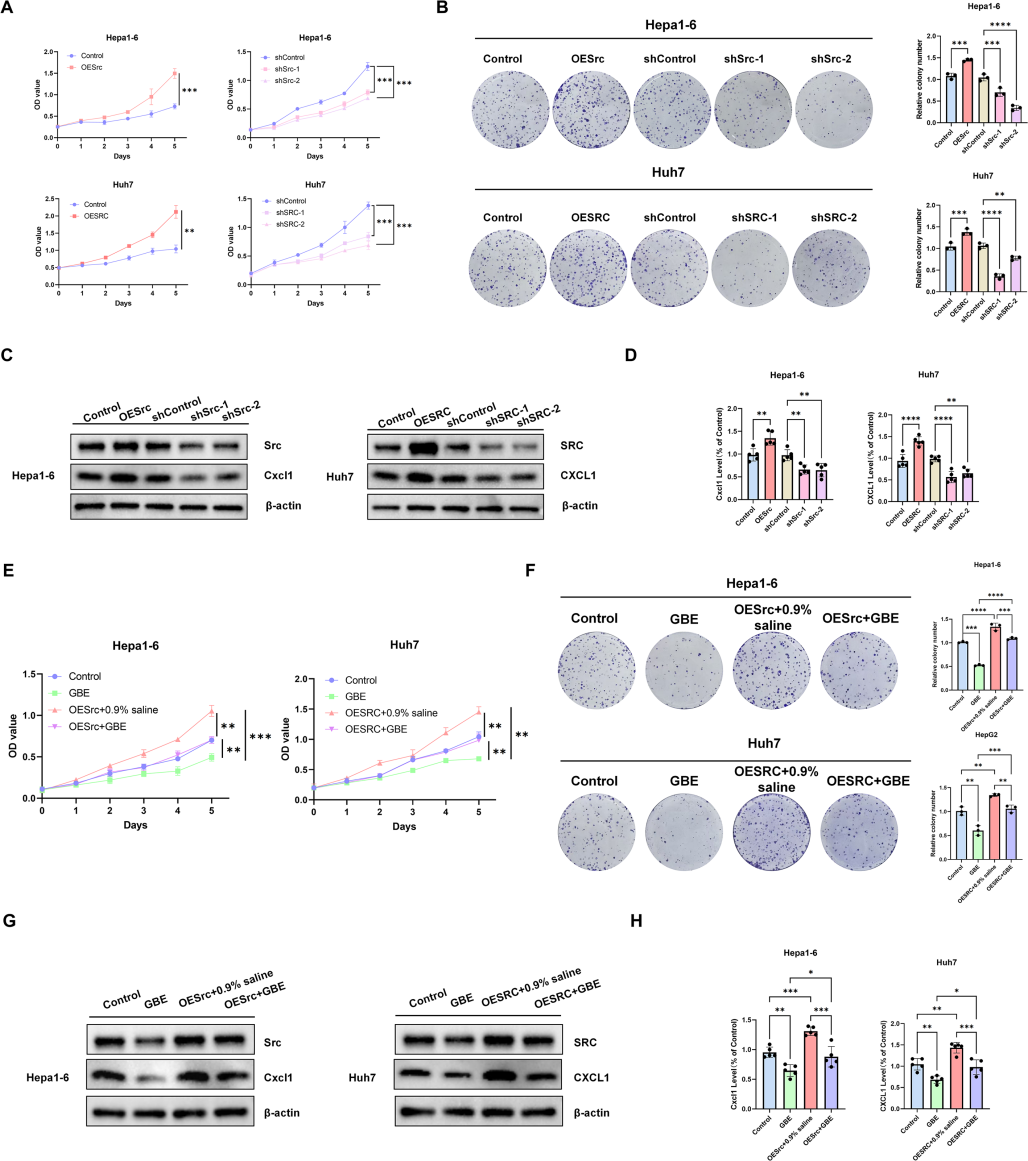
SRC acts as a core factor of GBE in suppressing HCC progression. **(A, B)** Proliferation of HCC cells with down-/up-regulation SRC expression were assessed by CCK8 **(A)** and colony formation assay **(B, C)** Western blot analysis of CXCL1 protein levels in HCC cells with down-/up-regulated SRC expression. **(D)** ELISA-based quantification of CXCL1 secretion in the culture supernatant of HCC cells following SRC modulation. **(E, F)** Proliferation of GBE-treated HCC cells with up-regulation SRC was assessed by CCK8 **(E)** and colony formation assays **(F, G)** Western blot analysis of CXCL1 protein levels in up-regulation SRC HCC cells treated with GBE. **(H)** ELISA-based quantification of CXCL1 secretion in the culture supernatant of HCC cells following GBE treatment and SRC modulation. All experiments were performed three times and data are presented as mean ± SD. *p< 0.05; **p<0.01; ***p<0.001; ****p<0.0001. HCC, Hepatocellular carcinoma; GBE, Ginkgo Biloba Extract; MDSCs, myeloid-derived suppressor cells; SRC, proto-oncogene tyrosine-protein kinase; CCK8, Cell Counting Kit 8; ELISA, Enzyme-Linked Immunosorbent Assay.

To examine how SRC contributes to HCC progression, we modulated Src expression in mouse Hepa1–6 cells. Growth curve and colony formation assays demonstrated that Src knockdown (shSrc) significantly inhibited HCC cell proliferation, whereas Src overexpression (OESrc) promoted proliferation (**Figures 5A, B**, upper panel). Western blot analysis revealed that OESrc enhanced Cxcl1 protein expression, whereas shSrc suppressed it (**Figure 5C**, left paner), suggesting Cxcl1 may function as a downstream effector molecule of Src. ELISA further confirmed that OESrc promoted Cxcl1 secretion, while its inhibition reduced secretion (**Figure 5D**, left paner). In critical rescue experiments, OESrc reversed GBE-mediated suppression of HCC cell proliferation (**Figure 5E**, left paner; **Figure 5F**, upper panel) and restored Cxcl1 expression (**Figures 5G, H**, left paner). Similar results were obtained with human Huh7 cells (**Figures 5A, B**, lower panel; **Figures 5C, D**, right paner; **Figure 5E**, right paner; **Figure 5F**, lower panel; **Figures 5G, H**, right paner). In summary, GBE inhibits HCC progression by suppressing SRC and subsequently reducing CXCL1 secretion, confirming SRC as a core regulatory target of GBE.

### 4.5 Elevated SRC expression correlates with poor prognosis and enhanced MDSCs recruitment in tumor tissues

**Figure 6:**
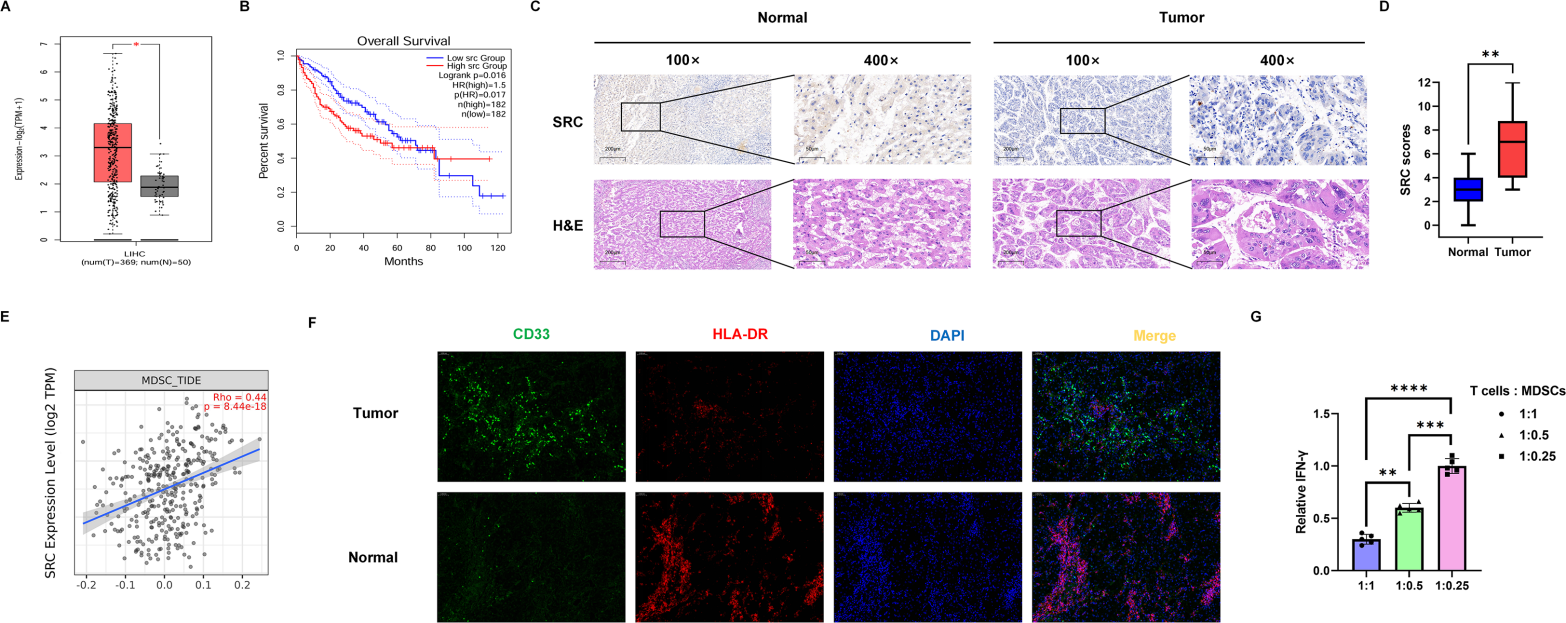
SRC is highly expressed in tumor tissues and is associated with poor prognosis as well as enhanced recruitment of MDSCs. **(A)**Expression of SRC based on GEPIA2 database in HCC. **(B)** Kaplan-Meier survival analysis for SRC expression and overall survival of patients with HCC. (n=182 per group, log-rank test p=0.016). **(C)** Immunohistochemistry analysis of SRC in tumor or adjacent normal tissues. **(D)** The score of SRC expression in 40 paired HCC tissue sections determined by Immunohistochemistry assay (**p < 0.01). **(E)** Correlation between SRC expression and MDSCs recruitment levels analyzed via TIMER2.0 database (p < 0.001). **(F)** Representative immunofluorescence images of MDSCs staining in tumor and adjacent normal tissues. CD33 (green) and HLA-DR (red) staining is shown. Cells exhibiting a CD33^+^HLA-DR^-^ phenotype are identified as MDSCs. Nuclei were counterstained with DAPI (blue). Scale bar, 50 μm. **(G)** T cell suppression assay using human tumor–derived MDSCs. Human T cells were co-cultured with MDSCs isolated from human tumor tissues at the indicated T cell-to-MDSCs ratios (1:1, 1:0.5, and 1:0.25). The 1:0.25 group was used as the control condition, in which MDSC-mediated suppression was minimal. Relative IFN-γ levels were measured. Data are presented as mean ± SD. Statistical significance was assessed by one-way ANOVA with *post hoc* tests, and significance levels are indicated in the figure. P values are indicated as **P < 0.01, ***P < 0.001, and ****P < 0.0001. HCC, Hepatocellular carcinoma; MDSCs, myeloid-derived suppressor cells; SRC, proto-oncogene tyrosine-protein kinase; GEPIA2, Gene Expression Profiling Interactive Analysis; IFN-γ, interferon-γ.

Based on the molecular mechanism by which SRC drives the malignant phenotype of HCC, we elucidated its prognostic value through multidimensional clinical analyses. The GEPIA2 database revealed that SRC expression was significantly higher in HCC tumor tissues compared to normal tissues (**Figure 6A**). The Kaplan-Meier survival analysis indicates that patients with low SRC expression (n=182) exhibited significantly better overall survival than those with high SRC expression (n=182) (p=0.016). (**Figure 6B**). A total of 40 pairs of HCC and adjacent non-tumorous tissues were examined. Immunohistochemical analysis revealed that SRC was overexpressed in HCC tissues. (**Figures 6C, D**). Analysis of the TIMER2.0 database indicated a significant positive correlation between SRC expression levels and MDSCs recruitment (p< 0.001) (**Figure 6E**). Immunofluorescence staining results demonstrated that, compared with normal liver tissues, MDSCs exhibited significant characteristic aggregation within the tumor microenvironment of HCC (**Figure 6F**). Consistently, T cells suppression assays showed that MDSCs isolated from human HCC tissues markedly inhibited T cells effector function (**Figure 6G**). These results demonstrate that SRC is aberrantly highly expressed in HCC tissues, and its expression intensity is closely associated with poor patient prognosis and remodeling of the immunosuppressive microenvironment.

## 5 Discussion

MDSCs are key regulators in the tumor microenvironment, inhibiting the anti-tumor functions of T cells and natural killer cells, which promotes immune escape and enhances tumor growth and spread (31). Recent studies have shown that various active components of TCM can enhance anti-tumor immune responses by modulating the function of MDSCs. For example, Eleutheroside A mitigates the immunosuppressive activity of MDSCs in gastric cancer by inhibiting their glycolytic pathway (32). Meanwhile, Siwu Decoction regulates MDSCs by inducing tumor cell necroptosis, thereby exerting anti-HCC effects (10). Additionally, components such as Ganoderma lucidum polysaccharides (33) and Ginsenosides (34) have been shown to modulate the differentiation and function of MDSCs, offering new strategies for tumor immunotherapy. Although GBE has been proven to exhibit anti-tumor activity, its specific regulatory mechanisms, particularly in immunomodulation, remain largely unclear. This study reveals that GBE exerts anti-HCC effects by targeting SRC expression to suppress the secretion of the chemokine CXCL1, thereby reducing the recruitment of MDSCs. Specifically, this study includes: (1) *in vitro* experiments demonstrating that GBE significantly suppresses HCC cell growth and decreases MDSCs recruitment within the tumor microenvironment; (2) network pharmacology analysis identifying SRC as a key target of GBE in HCC; and (3) experimental validation of the regulatory mechanism underlying the GBE–SRC–CXCL1–MDSCs signaling axis in HCC progression.

Numerous studies have demonstrated that GBE can modulate the immune microenvironment to influence disease progression. In neurological disorders, GBE promotes regulatory T cell differentiation by suppressing the HIF-1α/HK2 signaling pathway, thereby significantly improving outcomes in ischemic stroke (35). In metabolic diseases, GBE not only enhances serum immunoglobulin levels to boost antioxidant capacity in hepatic and intestinal tissues (36) but also ameliorates steatohepatitis and dyslipidemia by upregulating GLP-1 and β-catenin expression (37). Regarding hepatic protection, GBE exerts notable protective effects by suppressing inflammatory responses mediated by the TLR4-MyD88-NF-κB pathway and regulating T-cell homeostasis (38). Notably, although GBE has been shown to exert immunomodulatory and antitumor effects in lung cancer (39), its immunoregulatory mechanisms in HCC remain unclear. This study is the first to reveal that GBE exerts anti-HCC effects by specifically regulating MDSCs recruitment within the tumor microenvironment. However, the precise molecular mechanisms by which GBE regulates MDSCs recruitment remain to be elucidated through systematic experimental studies.

Network pharmacology has recently become a pivotal strategy for deciphering the complex mechanisms of TCM, significantly advancing the clinical translation of TCM compounds and their active constituents. For instance, network pharmacology has identified the PI3K/AKT pathway as a key target for Salvia miltiorrhiza in treating coronary heart disease (40); notably, its derivative, salvianolate, is now integrated into clinical treatment guidelines. Additionally, epimedium extract exerts anti-HCC effects by modulating ferritinophagy via the ERK/ULK1/NCOA4 signaling pathway, and its derivative, Icaritin, has been approved for clinical use as a therapeutic agent (41). In this study, network pharmacology analysis identified SRC as a potential key target of GBE against HCC. SRC, a pivotal member of the non-receptor tyrosine kinase family, has been shown to facilitate HCC progression by driving macrophage polarization (42). Moreover, the SRC/STAT3 signaling pathway regulates PD-L1 transcription, thereby modulating the immune evasion of HCC cells (43). In our study, molecular docking analysis revealed that the active components of GBE can spontaneously bind to SRC (binding energies < -5 kcal/mol). Experimental validation confirmed that GBE inhibits HCC progression by downregulating SRC, while SRC overexpression reverses this effect, identifying SRC as a key mediator of GBE’s action.

Building on these results, we further investigated the molecular mechanism by which GBE inhibits HCC progression through targeting SRC. Previous studies confirmed that GBE inhibits HCC progression by modulating SRC activity and suppressing MDSCs recruitment. In addition, numerous studies have indicated that SRC is one of the key proteins regulating the recruitment of MDSCs in the tumor microenvironment. Within this context, the SRC–JAK2 pathway promotes sustained STAT3 activation, leading to upregulation of CCL2 expression, which enhances MDSCs recruitment and facilitates tumor progression (44). Studies in preclinical settings show that targeting SRC markedly limits MDSCs buildup in head and neck cancer (45). In the present study, we confirmed that GBE inhibits SRC expression in HCC cells, leading to reduced CXCL1 secretion. CXCL1 is a key chemokine regulating MDSCs recruitment. Previous studies reported that SRC promotes immune escape in colon cancer by increasing CXCL1 secretion and MDSCs recruitment (46), consistent with our findings. These findings indicate that GBE suppresses HCC progression by targeting SRC, thereby lowering CXCL1 secretion and limiting MDSCs recruitment in the tumor microenvironment.

Accumulating evidence indicates that the recruitment of MDSCs into the tumor microenvironment plays a critical role in limiting the effectiveness of immune checkpoint inhibitors (ICIs). Elevated levels of MDSCs suppress cytotoxic T-cell function and reduce the therapeutic benefits of anti–PD-1/PD-L1 treatment. Several recent studies have shown that targeting immunosuppressive myeloid cells can restore antitumor immune responses, reshape the tumor immune microenvironment, and enhance sensitivity to ICIs (47). Moreover, SRC signaling contributes to immune escape and therapy resistance by regulating immune pathways and chemokine expression in the tumor microenvironment (48, 49). These effects may enhance the efficacy of combinatorial ICIs and epigenetic therapies in HCC (50). Collectively, these findings indicate that MDSCs recruitment and SRC-associated signaling create an immunosuppressive tumor microenvironment, reducing responsiveness to ICIs. By simultaneously targeting oncogenic signaling and tumor-associated immune suppression, GBE-based approaches may offer a promising strategy to enhance antitumor immunity.

This study is the first to systematically demonstrate that GBE targets the SRC/CXCL1 signaling axis, thereby inhibiting MDSCs recruitment and exerting potent anti-HCC effects. However, the precise molecular mechanism by which SRC transcriptionally regulates CXCL1—whether through direct phosphorylation of transcription factors or indirect modulation of epigenetic modifications at the CXCL1 promoter—remains to be elucidated. A limitation of this study is the use of a subcutaneous tumor model; orthotopic HCC models may better reflect the native hepatic microenvironment and will be addressed in future studies. Nonetheless, our findings on GBE-mediated regulation of the SRC/CXCL1 axis and its impact on MDSCs highlight a promising target and novel therapeutic strategy for HCC immunotherapy.

## 6 Conclusion

This study systematically uncovers a novel immune mechanism by which GBE inhibits HCC progression through the SRC/CXCL1 signaling axis to regulate MDSCs recruitment. Experimental results demonstrate that GBE significantly suppresses SRC expression, downregulates CXCL1 secretion, and consequently reduces MDSCs recruitment in the tumor microenvironment. Although the precise molecular mechanism by which SRC transcriptionally regulates CXCL1 remains to be elucidated, these findings provide a theoretical basis for the potential application of GBE in HCC immunotherapy and offer experimental support for therapeutic strategies targeting the SRC/CXCL1/MDSCs axis.

## Data Availability

The original contributions presented in the study are included in the article/**Supplementary Material**. Further inquiries can be directed to the corresponding authors.
