# Supplementary material for: Ginkgo biloba extract suppresses hepatocellular carcinoma progression by inhibiting the recruitment of myeloid-derived suppressor cells through reduced CXCL1 secretion via SRC downregulation
Source: Front Immunol. 2026 Jan 19;16:1750890. doi: 10.3389/fimmu.2025.1750890 (PMC12862084; doi:10.3389/fimmu.2025.1750890)
Supplement: Supplementary file 2 [file Table1.doc]

**Supplementary Table**

**Table S1. Chemicals.**

| **REAGENT or RESOURCE** | **SOURCE** | **IDENTIFIER** |
| --- | --- | --- |
| **Chemicals, Peptides, and Recombinant Proteins** |  |  |
| Ginkgo biloba extract | KKL | Cat# KP1532 |
| Normal Saline, 0.9% | Solarbio | Cat# IN9000 |
| Penicillin-streptomycin | ThermoFisher | Cat# 15140148 |
| Dulbecco's Modified Eagle Medium | ThermoFisher | Cat# 11965092 |
| Fetal Bovine Serum | ThermoFisher | Cat# A5256701 |
| Trypsin-EDTA Solution，0.25%(without Phenol Red) | Solarbio | Cat# T1300 |
| Phosphate-Buffered Saline | Solarbio | Cat# P1020 |
| Trypan Blue Stain Solution，0.4% | Solarbio | Cat# C0040 |
| Pentobarbital sodium salt | Bei Jing Think-Far Technology | Cat# P3761 |
| Human ELISA CXCL1 Kit | R and D Systems | Cat# DY275, RRID:AB_3675361 |
| Mouse ELISA Cxcl1 Kit | R and D Systems | Cat# DY453, RRID:AB_3675362 |
| 4% Paraformaldehyde Fix Solution | Beyotime | Cat# P0099 |
| Crystal Violet Ammonium Oxalate Solution | Solarbio | Cat# G1062 |
| Lipofectamine 3000 Reagent | ThermoFisher | Cat# L3000015 |
| Goat Serum | Beyotime | Cat# C0265 |
| Nonfat Dry Milk | Lab Scientific | Cat# M0841 |
| Triton X-100 | Bio-Rad | Cat# 161-0407 |
| Tween 20 | Fisher BioReagents | Cat# BP337-100 |
| Immun-Blot PVDF Membrane | Bio-Rad | Cat# 162-0177 |
| Cell Counting Kit-8 | YEASEN | Cat# 40203ES60 |
| Super ECL Detection Reagent ECL | YEASEN | Cat# 36208ES60 |
| Ampicillin | Invitrogen | Cat# Q60120 |
| Protease Inhibitor Cocktail | Sigma-Aldrich | Cat# P8340 |
| ClonExpress Ultra One Step Cloning Kit | Vazyme | Cat# C115 |
| DAPI | ThermoFisher | Cat# 62248 |
| 180 kDa Prestained Protein Marker | Vazyme | Cat# MP102-01 |
| Polybrene | Beyotime | Cat# C0351 |
| Hematoxylin Staining Solution | Beyotime | Cat# C0107 |
| Eosin Staining Solution | Beyotime | Cat# C0109 |
| Acid Alcohol Slow Differentiation Solution | Beyotime | Cat# C0161 |
| Bluing Solution | Solarbio | Cat# G1866 |
| Maxvision™ HRP-polymer anti-mouse/anti-rabbit IHC kit. | Maxim | Cat# KIT-5020 |
| Vectastain Elite ABC-HRP Kit | Vector lab | Cat# PK-6100 |
| TSAPLus triple fluorescent staining kit | Servicebio | Cat# G1226-50T |
| Mouse Interferonγ,IFN-γ ELISA Kit | Cusabio Biotech | Cat# CSB-E04578m-IS |
| Human IFN-γ(Interferon Gamma) ELISA Kit | Elabscience | Cat# E-EL-H0108c |

**Table S2. Antibodies.**

| **REAGENT or RESOURCE** | **SOURCE** | **IDENTIFIER** | | **DILUTION** | |
| --- | --- | --- | --- | --- | --- |
| **Antibodies** | | | | | |
| RAT ANTI MOUSE Gr-1 Mouse | Bio-Rad | Cat# MCA2387T | RRID:AB_2115659 | IF: 1:200 |  |
| Vector M.O.M. Kit (Mouse-On-Mouse Immunodetection) | Vector Laboratories | Cat# FMK-2201 | RRID:AB_2336834 | IF: 1:200 |  |
| Alexa Fluor® 647 Anti-CD11b antibody Rabbit | Abcam | Cat# ab307523 | RRID: AB_3717315 | IF: 1:100 |  |
| Anti-CXCL1/GRO alpha antibody Rabbit | Abcam | Cat# ab86436 | RRID:AB_2087574 |  | WB: 1:1000 |
| Anti-SRC antibody Rabbit | Abcam | Cat# ab47405 | RRID:AB_870739 |  | WB: 1:1000 |
| Anti-beta Actin antibody Rabbit | Abcam | Cat# ab115777 | RRID:AB_10899528 |  | WB: 1:200 |
| Anti-Rabbit IgG H&L (HRP) Goat | Abcam | Cat# ab6721 | RRID:AB_955447 |  | WB: 1:10000 |
| Anti-CD45 Rabbit pAb | Servicebio | Cat# GB11066-100 | RRID: AB_3717604 |  | WB:1:200 |
| Anti-CD11b Rabbit pAb | Servicebio | Cat# GB115735-100 | RRID:AB_3717603 |  | WB:1:200 |
| Anti-CD45 Rabbit pAb | Servicebio | Cat# GB113885-100 | RRID: AB_3717605 |  | WB:1:200 |
| Anti-human CD33 Mouse | Trillium Diagnostics | Cat# CD33-251P | RRID:AB_2333086 | IF: 1:100 |  |
| Alexa Fluor® 488-AffiniPure Goat Anti-Mouse IgG (H+L) (min X Hu,Bov,Hrs,Rb,Sw Sr Prot) antibody Goat | Jackson ImmunoResearch Labs | Cat# 115-545-146 | RRID:AB_2307324 | IF: 1:100 |  |
| Mouse Anti-Human HLA-DR (HLA-DRA) Monoclonal, Unconjugated, Clone 5f62 Mouse | LSBio | Cat# LS-C24521-100 | RRID:AB_901974 | IF: 1:100 |  |
| Alexa Fluor® 555-conjugated AffiniPure Goat Anti-Mouse IgG H&L Goat | MedChemExpress | Cat# HY-P81006 | RRID:AB_3103151 | IF: 1:100 |  |
