# Supplementary material for: Ginkgo biloba extract suppresses hepatocellular carcinoma progression by inhibiting the recruitment of myeloid-derived suppressor cells through reduced CXCL1 secretion via SRC downregulation
Source: Front Immunol. 2026 Jan 19;16:1750890. doi: 10.3389/fimmu.2025.1750890 (PMC12862084; doi:10.3389/fimmu.2025.1750890)

Figure 5C

Hepa1-6

Src

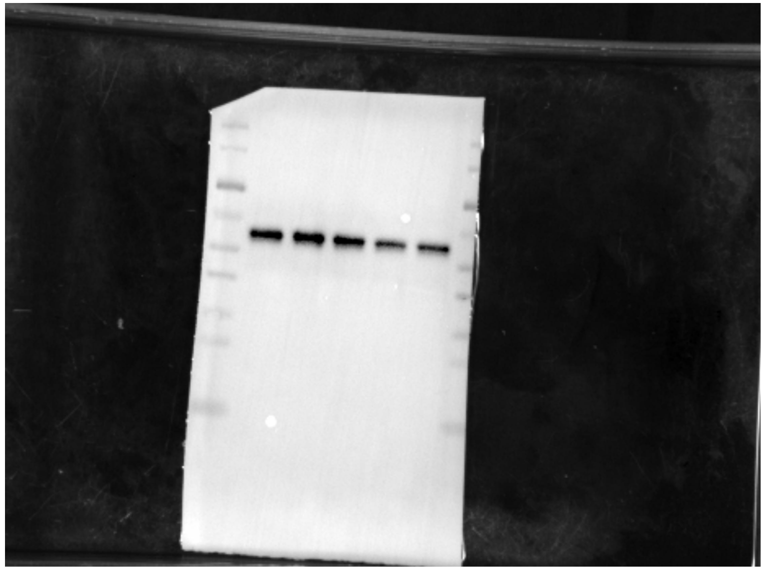

Cxcl1

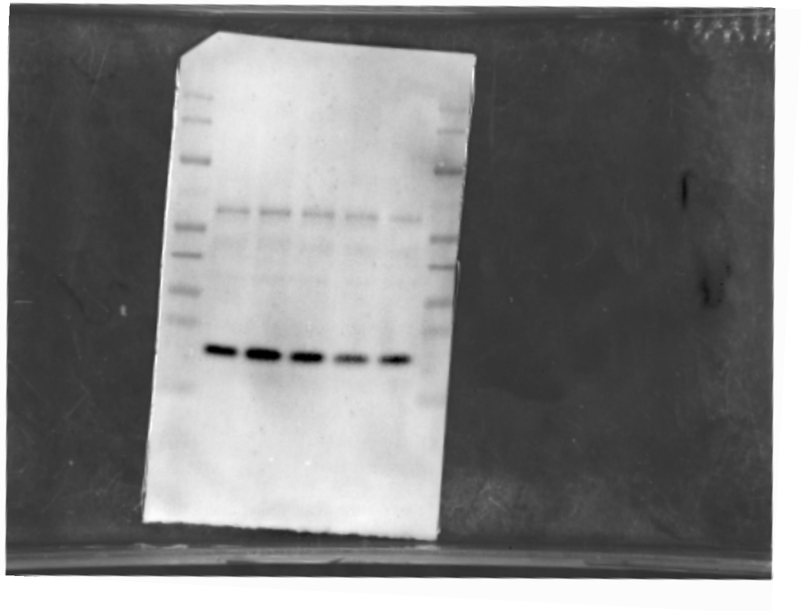

β-actin

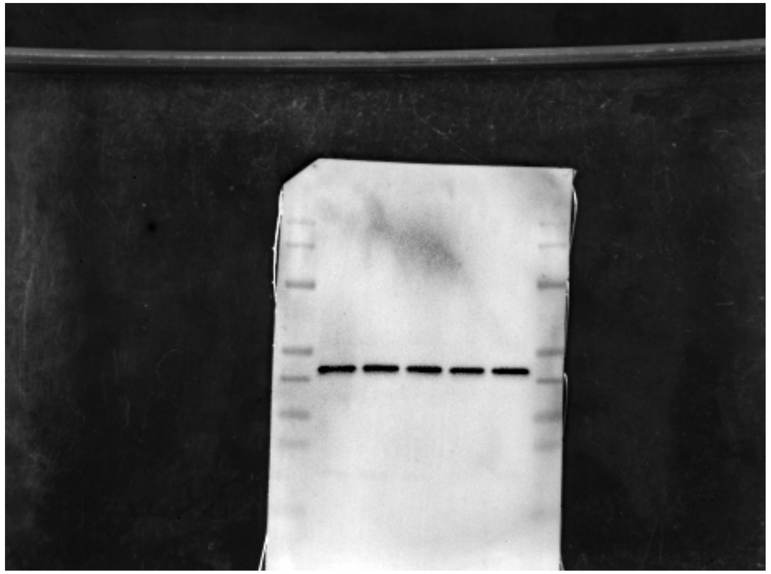

Figure 5C

Huh7

SRC CXCL1 β-actin

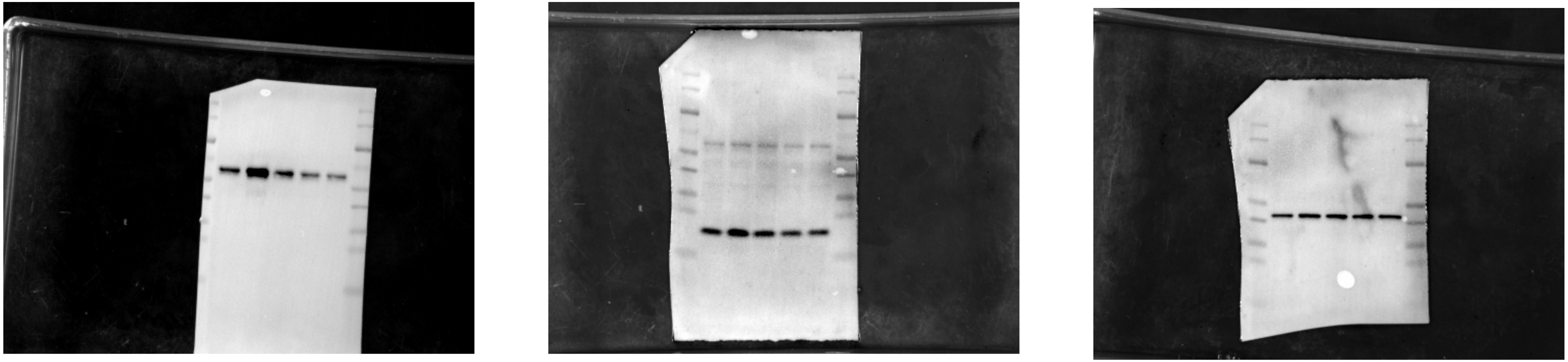

Figure 5G

Hepa1-6

Src

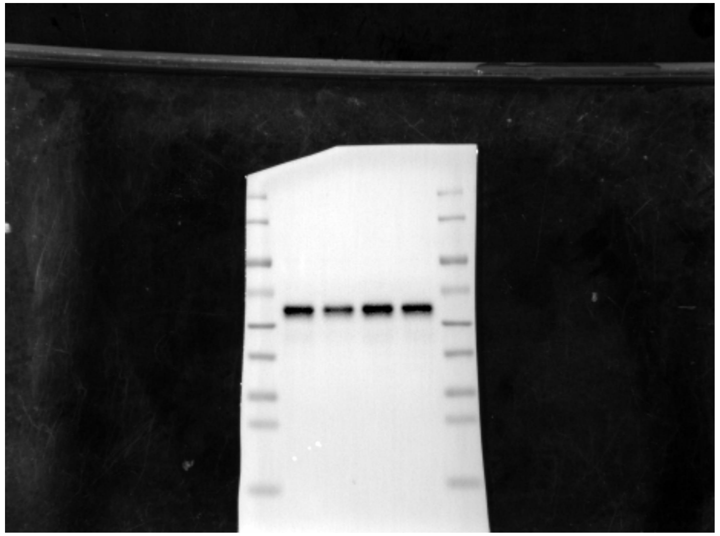

Cxcl1

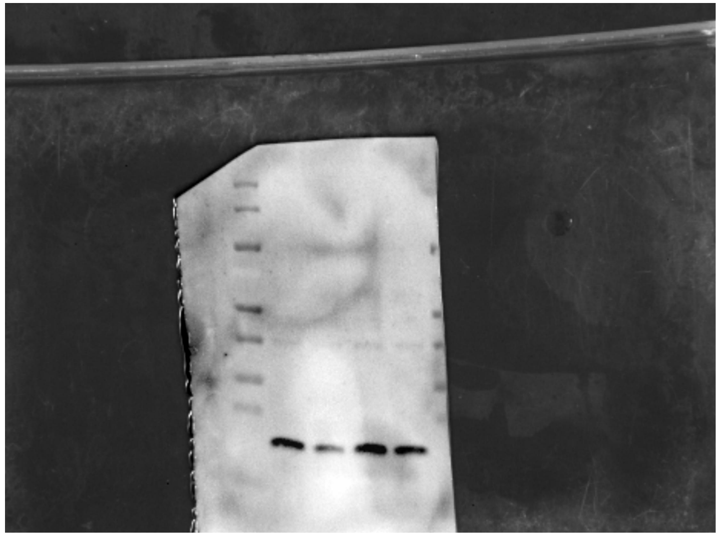

β-actin

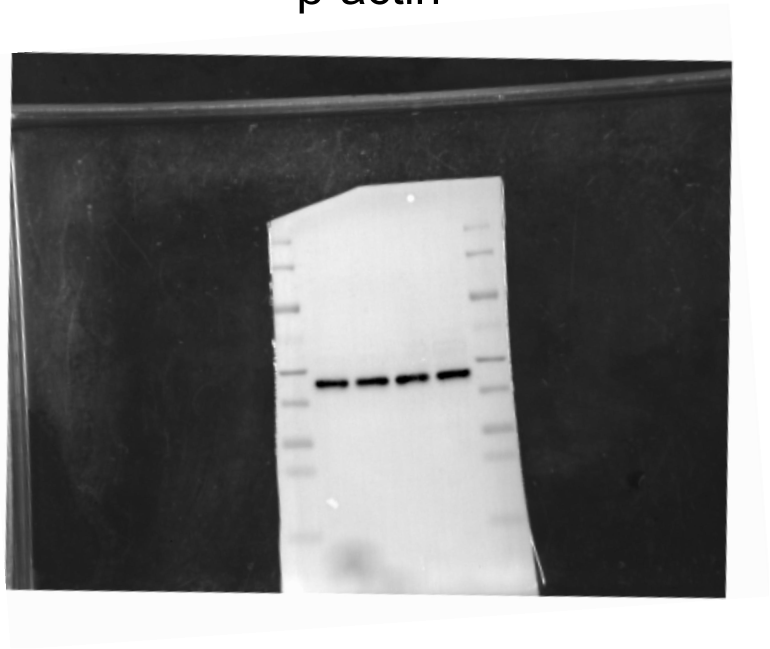

Figure 5G

Huh7

SRC

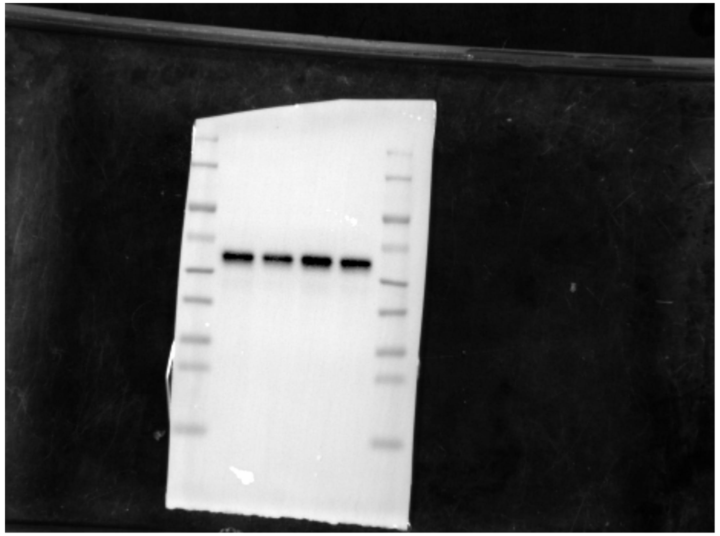

CXCL1

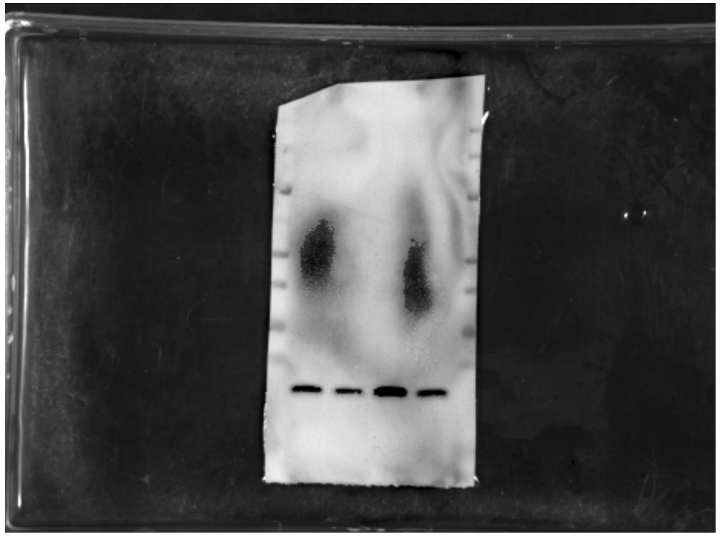

β-actin

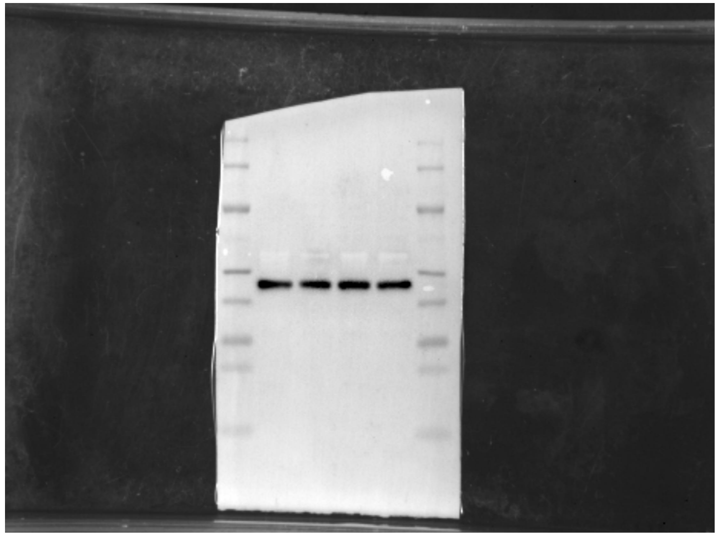

Supplement: Supplementary file 3 [file Presentation1.pdf]
